# Mentor’s Perspective on Structured Clinical Mentoring in the Arab Context.

**DOI:** 10.12688/f1000research.177515.2

**Published:** 2026-04-30

**Authors:** Jacqueline Maria Dias, Semiyu Adejare Aderibigbe, Mini Sara Abraham, Abdullah A. A. Sankar, Mohammed M. M. Mansour, Mohammed Gamil Awadh, Yousseif Mohamed Yousseif Mohamed Awadalla Elsatary, Mohammed B. A. Ballour, Muhammad Arsyad Subu, Nabeel Al Yateem, Fatma Refaat Ahmed, Al-Hasan Mohammed Abdullah Al-Azzani, Edric Paul Dias, Richard Mottershead

**Affiliations:** 1 Department of Nursing, College of Health Sciences, University of Sharjah, Sharjah, United Arab Emirates; 2 College of Public Policy, University of Sharjah, Sharjah, United Arab Emirates; 3 College of Science, Arts and Letters, University of Michigan, Michigan, USA; 4 Behavioural Science Institute, SEHA, Al Ain, United Arab Emirates, Sakina, Al Ain, United Arab Emirates; 5 College of Nursing, University of Baghdad, Baghdad, Iraq

**Keywords:** Mentor, clinical, mentorship, nursing, qualitative methods, United Arab Emirates

## Abstract

**Background:**

Mentorship is essential in nursing education to foster clinical skills, critical thinking, and professional identity. Despite extensive research on mentorship, few studies have addressed its role in the Arab cultural context. This study explored nursing mentors’ clinical learning experiences through mentorship within the cultural context of the United Arab Emirates (UAE).

**Methods:**

A qualitative approach was employed, involving 20 mentors supervising fourth-year nursing students during their final clinical placement at a semi-public university in the UAE. The placement occurred from January to May 2024, as part of the Consolidation of Practice course, comprising 240 hours of clinical training. Structured and semi-structured in-depth interviews were conducted with the participants, and the data were transcribed verbatim. To analyse the data, an inductive thematic approach was adopted, and some data were quantified for additional insights.

**Results:**

Four main themes emerged regarding the benefits of structured mentoring within the cultural context: critical for practical training, confidence building, bridging theory and practice and mutual learning. The essential mentoring skills identified were effective communication, patience, and understanding. Structured mentoring frequency positively influenced students’ clinical learning. The strengths of the structured mentorship included exposure to real-life scenarios, improved communication, and the development of practical skills. Opportunities for improvement included increasing mentor–student interactions, enhancing the programme’s structured nature, and integrating technological tools. The mentors recommended reassessing mentorship duration, increasing hands-on clinical exposure, strengthening mentor collaboration, and promoting student accountability.

**Conclusion:**

Effective mentorship in nursing education in the UAE requires integrating theory and practice, clear communication, and leveraging technology to overcome barriers. Strengthening structured mentor–student interactions through focused workshops and refined programme structures can bridge educational gaps. Such enhancements can enable nursing students to develop into competent and confident healthcare professionals, who are familiar with culturally informed mentorship practices.

## 1. Introduction

Clinical education is the cornerstone of nursing curricula and provides essential experiential learning opportunities for students in authentic healthcare settings. ^1^ These practical experiences are crucial for bridging the gap between theoretical knowledge and real-world applications and ensuring that nursing graduates are adequately prepared for the complexities and evolving demands of contemporary clinical practice. ^2^ The effectiveness of clinical education significantly depends on structured mentorship programmes, which facilitate skill acquisition, clinical decision-making capabilities, and professional confidence. ^2, 3^

Globally, mentorship is recognised as a pivotal component of nursing education that significantly affects students’ clinical competence, professional identity, and preparedness for independent practice. ^3, 4^ However, its implementation and effectiveness in Arab cultural contexts, particularly in the United Arab Emirates (UAE), require further exploration. Mentorship in these settings often incorporates culturally sensitive practices designed to accommodate diverse student needs and enhance students’ clinical experiences. Effective mentorship in the UAE context fosters environments that empower students, enabling them to develop the technical skills and culturally responsive care capabilities essential for high-quality patient care. ^2, 5^

Effective clinical mentorship in the UAE involves robust collaboration between academic nursing programmes and healthcare institutions. Experienced clinical mentors guide students by assisting them in translating theoretical concepts into clinical practice through structured engagement and hands-on training. ^2, 5^ Such collaboration ensures coherence between educational objectives and clinical experiences and promote skill development, critical thinking, and confidence among students.

Despite these recognised benefits, mentorship faces challenges, such as identifying suitable mentors, managing clinical workloads, and addressing mentors’ preparedness. ^4, 6^ Innovative solutions, including virtual and metaverse-based mentoring programmes, have emerged as promising alternatives that offer immersive and interactive experiences capable of enhancing mentorship accessibility and effectiveness. ^4^

This study sought to deepen our understanding of the role of mentorship in skill acquisition, confidence building, essential mentoring skills, and structured interactions within clinical learning environments. By exploring these areas, this study provided strategic insights for enhancing the effectiveness of mentorship practices, thus contributing significantly to nursing education and clinical practice outcomes in the Arab region. To achieve these goals, this study addressed the following research questions:

1. How does mentorship influence the practical skills and confidence of nursing students?
2. What are the critical mentoring skills necessary for effective learning outcomes in clinical settings?
3. How do the frequency and structure of mentoring interactions affect the learning outcomes of nursing students?
4. How can strengths and opportunities be leveraged to enhance the mentoring scheme in this study’s clinical setting?

## 2. Literature review

### 2.1 Cultural influences on mentorship

The cultural landscape of the UAE significantly shapes nursing mentorship practices. Cultural values such as communal solidarity, family-like relational dynamics, and mutual respect are integral to mentorship in UAE healthcare settings, shaping mentor and mentee interactions. The mentorship relationship in this context goes beyond technical skills and involves deep interpersonal connections that reflect broader cultural values. Students who receive mentorship aligned with cultural norms experience enhanced adaptation to clinical environments and professional confidence. ^5^ These cultural influences underline the necessity for culturally responsive mentorship frameworks, which are essential for the successful integration of nursing students into diverse clinical environments.

### 2.2 Diversity and mentorship in healthcare

Globally, healthcare systems, including those in the UAE, increasingly operate in multicultural environments that demand the effective integration of diverse nurses into cohesive clinical teams. Effective mentorship, complemented by robust peer support, substantially assists new nurses from diverse backgrounds in navigating workplace challenges and enhances their overall professional development. Given the UAE’s culturally diverse workforce, healthcare institutions must foster inclusive mentorship environments that recognise and address cultural and linguistic differences. ^6^ Implementing structured mentorship programmes that emphasise cultural sensitivity is vital for developing cohesive clinical teams and supporting nurses’ professional transitions and continued growth.

### 2.3 Structural barriers to mentorship

Organisational structure and workload demands heavily influence the frequency and effectiveness of mentorship interactions. Rigid scheduling and demanding clinical workloads create significant barriers to effective mentor–student engagement, limiting the consistency and quality of mentorship interactions. These structural barriers necessitate organisational interventions such as protected mentorship time and adjusted workloads to facilitate meaningful mentor–mentee relationships. ^6^ Without addressing these systemic issues, the full benefits of mentorship programmes remain unrealised, highlighting the importance of management support in ensuring effective mentorship practices.

The effectiveness of mentorship depends on mentor selection processes. Ideal mentors should possess clinical expertise, strong communication skills, and the ability to foster supportive professional relationships. Effective mentors typically demonstrate high levels of empathy, problem-solving ability, and leadership skills, which significantly impact mentees’ skill development and professional confidence. ^7– 9^ Mentor proximity and availability and mentees’ perceptions of their mentors’ professional competence and support are critical factors that influence successful mentorship outcomes. ^10^ These insights suggest that healthcare institutions and academic programmes must develop rigorous mentor selection criteria that emphasise interpersonal skills, cultural competence, and professional expertise to enhance mentorship effectiveness. ^11, 12, 29^

### 2.4 Mutual learning and mentorship

Mentorship is increasingly being recognised as a reciprocal process that benefits both mentors and mentees. The literature underscores mentorship as a dynamic interaction in which experienced nurses enhance their professional and personal growth through reflective practice and teaching responsibilities, which benefits mentees through enriched clinical guidance. ^13, 14^ Experienced mentors report personal and professional growth through reflective practice and continuous skill refinement, demonstrating the multidirectional benefits of mentorship. ^13^ These findings emphasise the potential of mentorship programmes to transform clinical environments into collaborative learning communities and foster professional development for mentors and mentees. ^15– 18, 30^

### 2.5 Bridging theory and practice

Mentorship effectively bridges the gap between theoretical knowledge and clinical application, facilitating nursing students’ development of practical skills and clinical judgment. ^19^ Mentors play a pivotal role in translating classroom theory into practical skills through individualised instruction and feedback, significantly enhancing students’ readiness for clinical practice. ^20^ This bridging role is essential in nursing education as it promotes critical reflection and a deeper understanding of nursing roles and responsibilities, ultimately contributing to higher standards of patient care and professional fulfilment. ^21, 22^

### 2.6 Technology integration in mentorship

Emerging technological solutions, such as virtual reality and digital learning platforms, offer innovative approaches to mentorship by enhancing teaching capabilities and providing flexible and accessible learning opportunities. Integrating technology into mentorship programmes can effectively overcome traditional mentorship limitations such as scheduling challenges and limited mentor availability. ^4^ Additionally, technological advancements enable tailored mentorship approaches, offering individualised support and feedback mechanisms that significantly enhance learning outcomes. ^23^ Thus, technological integration represents a critical opportunity for advancing mentorship effectiveness, particularly in resource-constrained environments.

### 2.7 Implications of the literature reviewed

Drawing on the reviewed literature, effective mentorship practices require structured support from nursing management, comprehensive mentor training programmes, culturally informed mentorship strategies, and innovative technological solutions. ^24^ Consequently, healthcare, and academic institutions in the UAE should strategically implement evidence-based practices to facilitate enriched mentor–mentee relationships and enhance clinical education outcomes. Such improvements are expected to foster inclusive clinical learning environments, increase retention rates among nursing professionals, and ensure ongoing professional growth within the culturally diverse healthcare settings that characterise the UAE. However, the literature indicates the need for additional data derived from the perspectives of key stakeholders to effectively understand and optimise mentoring experiences. Therefore, this study specifically addressed this gap by focusing on mentors’ insights into student learning outcomes in clinical education contexts that employ structured mentoring within the unique cultural environment of the UAE.

## 3. Materials and methods

### 3.1 Study design

This study used a qualitative approach, employing semi-structured interviews and qualitative content analysis to explore mentors’ perceptions and experiences in nursing education. Content analysis, an established interpretive technique, facilitates the systematic coding and categorisation of qualitative data and identifies patterns and thematic structures within textual data. ^25^ This allows for a comprehensive examination and interpretation of similarities, differences, and relationships within and across textual information, thus ensuring reliable and detailed insights into mentorship experiences.

### 3.2 Setting and participants

The research was carried out among mentorship programs in hospitals in the UAE where mentors oversaw clinical practice for undergraduate nursing students during the final clinical practicum. The selection process for the participants was done by means of purposive sampling to include mentors with pertinent clinical and mentoring experience. The inclusion criteria comprised clinical expertise, willingness to participate, effective communication abilities, and active mentoring roles with undergraduate students during their final clinical practicum. A total of 20 mentors were recruited based on their mentorship experience, clinical expertise, and commitment to student guidance. No eligible mentor declined to participate or later withdrew once the study commenced.

### 3.3 Research instrument

Data were collected using a semi- structured interview guide based on an extensive literature review focusing on mentorship, nursing students, mentors, and mentees. The interview questions underwent expert validation by two additional nursing faculty members experienced in mentorship research. The structured interview guide comprised of three main sections.

Section 1: Importance of Mentorship

• Importance and benefits of mentorship.
• Preferred communication styles.
• Essential skills required for effective mentorship.

Section 2: Effects on Student Learning

• Frequency and structure of mentorship interactions.
• Perceived impact on student learning and clinical competence.

Section 3: Program Evaluation (SWOT Perspective)

• Identification of strengths and weaknesses, of the existing mentorship scheme.
• Opportunities for enhancement and potential challenges
• Recommendations for improving mentorship effectiveness.

### 3.4 Data collection

The data were collected using semi-structured face-to-face interviews conducted between January and May 2024 in private, quiet locations within the participating hospital settings while ensuring confidentiality and comfort for participants. Each interview lasted approximately 30–45 minutes allowing for sufficient depth to explore mentors experiences and perspectives. The interviews were conducted by an experienced qualitative researcher in nursing education with prior experience in mentorship research who facilitated open and candid discussions regarding the mentors’ experiences, perceptions, and recommendations. The interviews were digitally recorded, transcribed verbatim, and supplemented with detailed field notes and reflective memos taken by the research team. The point of data saturation was described as the moment when there were no more codes and themes discovered by subsequent interviews. It was assessed collaboratively by the team of researchers in the process of analysis of data. No further codes were discovered after about 17 interviews, and three more interviews were conducted to ensure the achievement of saturation, thus a total of 20 interviews.

### 3.5 Data analysis

Thematic analysis was done using the method of reflexive thematic analysis as provided by Braun and Clarke. ^26^ This was adopted in order to be able to conduct a thorough analysis of the experiences of the mentors within the culture of the UAE. Thematic analysis involves six steps including (i) familiarization with the data through repetitive readings of transcripts; (ii) creating the initial codes; (iii) developing themes through pattern identification; (iv) refining the themes; (v) naming the themes and finally (vi) developing the final report.

Coding was done inductively, beginning from codes which arose out of the data set itself. Two researchers from the research team independently analyzed the transcripts and generated codes. The codes were discussed and revised iteratively until an agreed upon set of codes was obtained. Coding was an iterative process that entailed consistent comparison of data in order to generate codes and find similarities and differences in the codes Theme generation was carried out inductively where there was no guidance from any framework. Themes were allowed to emerge out of the data set. Iteration between data and interpretation were constantly made in the analysis to ensure thoroughness and coherence of the analysis. Discussions were held among the research team to refine themes further. Participant checking was not done. However, methodological rigor was ensured through group discussions and extensive use of verbatim quotes.

### 3.6 Ethical considerations

The University of Sharjah Research Ethics Committee (REC) approved this study (Number: Reference Number: REC 24–01–02-01-S). The participants received comprehensive explanations of the study’s purpose, procedures, risks, and voluntary participation and provided written informed consent. The interviews were conducted privately in hospital settings to ensure confidentiality and participant comfort. Confidentiality was maintained by assigning random alphanumeric codes (e.g., M1 for Mentor 1) to the participants and securely storing the digital recordings and transcripts on password-protected devices accessible only to the research team. The participants were informed of the researchers academic role and the study purpose and were assured that their involvement or responses would not affect their roles as clinical mentors. Upon completing the analysis, the digital data were securely stored and scheduled for future secure destruction.

## 4. Results and discussion

In this section, the insights derived from the collected data are presented and analysed. The findings illustrated key themes and addressed the specific research questions, providing comprehensive insights into the mentorship experiences and outcomes in the clinical setting.

### 4.1 How does mentorship influence the practical skills and confidence of nursing students?

To determine the influence of the mentorship scheme on nursing students’ development of practical skills and confidence, we asked mentors to clarify the benefits of the initiative and justify their views. From the analysed data, two main themes emerged as follows, along with supporting vignettes.

*4.1.1 Critical for practical learning*

The participants underscored the critical role of mentoring in providing hands-on experience and practical learning. This sentiment was articulated as follows:

*“Mentoring allows them to practice and learn the right procedures.” (M1)*

*“The mentor has to be a role model so that the students can learn practical experiences and procedures from the mentor’s teachings.” (M2)*

These responses highlighted the necessity for practical hands-on learning experiences provided through mentoring, which are essential for developing clinical skills in nursing students.

*4.1.2 Confidence building*

The participants also highlighted the role of mentoring in building student confidence and competence. This view was exemplified in the following text:

*“It helps students transition from theory to clinical practice and builds their confidence and competence.” (M4)*

*“Provides field experience, exposes students to clinical settings, and helps them gain confidence.” (M3)*

Building confidence is a critical aspect of mentoring that helps students become more competent and self-assured in their clinical practice. The unanimous agreement among mentors regarding the importance of mentoring reflects its perceived essential role in nursing education. This suggests that mentoring is not just beneficial but also vital for the professional development of nursing students.

Given that the participants substantially alluded to the importance of mentoring, we asked them to clarify their views and thematically analysed the data, with the following two themes emerging.

*4.1.3 Bridging theory and practice*

The participants clarified how mentoring helps students apply theoretical knowledge in practical settings to justify their view that mentoring is important. Below are some examples of these explanations:

*“It provides clarity on procedures and how to deal with patients from an experienced person.” (M5)*

*“To convert theoretical knowledge into practical skills.” (M14)*

Mentoring is important for transforming theoretical knowledge into practical skills, which is essential for effective nursing practice.

*4.1.4 Mutual learning*

Interestingly, the participants acknowledged the role of mentoring in fostering mutual learning for mentors and students in terms of professional learning development and growth. This perspective was described as follows:

*“Both the mentor and the student can learn from each other.” (M16)*

*“Mentoring allows them to practice and learn the right procedures.” (M11)*

From the data analysed, it was evident that bridging the gap between theory and practice and the mutual benefits for both mentors and mentees were the primary reasons for the importance of mentoring. These aspects highlighted the practical and relational dimensions of effective mentoring.

The findings demonstrated that mentorship is a critical component of nursing education and significantly enhances students’ practical skills and confidence. The participants strongly emphasised that mentorship provides crucial hands-on training, facilitating the transition from theoretical knowledge to practical application. Mentors serve as role models who demonstrate clinical procedures and decision-making processes. These results align with the literature that emphasises experiential learning as central to developing professional competence and confidence among nursing students. ^27^ Moreover, practical exposure directly enhances clinical judgment and patient safety. Thus, institutions should ensure structured and consistent mentorship experiences that emphasise the development of practical skills. Enhanced training for mentors via procedural demonstrations and role modelling is necessary to improve students’ clinical preparedness and confidence.

### 4.2 What are the critical mentoring skills necessary for effective learning outcomes in clinical settings?

As clinical and workplace learning through mentoring engagement is required for nursing students, it is essential to explore the participants’ views on the required mentoring skills. Through their responses, the participants revealed two critical themes.

*4.2.1 Communication skills*

The participants overwhelmingly highlighted the importance of effective communication in mentoring. Below are some examples that support this notion:

*“Proper communication skills, the ability to pace down work to teach students effectively.” (M12)*

*“Good listener, good communication skill, critical thinking.” (M11)*

Effective communication is fundamental to mentoring and facilitates clear instruction, feedback, and understanding between mentors and mentees.

*4.2.2 Patience and understanding*

The participants also acknowledged the need for patience and an understanding of mentoring relationships. This sentiment was exemplified as follows:

*“Understanding the student’s point of view, needs, and objectives, and improving one’s skills to meet student needs.” (M7)*

*“Patience, understanding students’ needs, knowing what to teach.” (M17)*

The analysis consistently highlighted communication skills and patience, indicating their critical role in effective mentoring. These skills are necessary for fostering a supportive learning environment and ensuring clear and effective guidance.

The critical mentoring skills included effective communication, patience, and understanding. The participants underscored communication as essential for clear instruction, feedback, and fostering trust in mentor–mentee relationships. In culturally diverse healthcare environments such as the UAE, effective communication helps bridge potential cultural and linguistic gaps, facilitating more productive interactions. The literature consistently indicates that communication skills are fundamental to successful mentorship, particularly in multicultural settings. ^12^ Patience and empathy are equally vital, allowing mentors to adjust their teaching to individual student needs and ensuring positive learning outcomes. By implication, specialised mentor training programmes are necessary, which focus on communication strategies, cultural competency, and emotional intelligence to optimise mentorship effectiveness within culturally diverse settings.

### 4.3 How does the frequency and structure of mentoring interactions affect the learning outcomes of nursing students?

To analyse the mentors’ responses and address this research question, we identified the common themes related to the three specific questions posed. The themes were grouped according to their respective questions. To provide a structured overview of the participants’ responses, descriptive counts of commonly reported perspectives are presented alongside the qualitative findings. As this study is qualitative in nature, numerical summaries are presented as descriptive counts are intended to complement, rather than quantify, the thematic analysis.

Table 1 shows that most mentors ( *n*=12) preferred daily interactions with students, indicating a belief in the importance of consistent and frequent contact for effective mentoring. A significant proportion( *n*=7) found weekly or biweekly interactions to be sufficient, suggesting some flexibility in effective mentoring practices. Only ( *n*=1) adjusted the frequency based on specific needs, highlighting the need for tailored approaches.

**Table 1.** Preferred frequency of interaction.

Frequency	Number of mentors ( *n*=20)
Daily	12
Weekly or Biweekly	7
Variable	1

Table 2 shows that half of the mentors ( *n*=50) provided specific examples of improvements in clinical competence, reinforcing the tangible benefits of mentorship. Following this category, six of mentors noted general improvements, which indicated positive outcomes but with less specificity. One mentor highlighted the difficulty in assessing improvements due to limited exposure or student engagement.

**Table 2.** Observed improvements in clinical competence.

Observation	Number of mentors ( *n*=20)
Provided Specific Examples	10
Reported General Improvement	6
Noted Contextual Factors	1
No Clear Response	3

To gain deeper insight into how the number of mentors influences outcomes, we analysed the relationship between the number of mentors and their respective impacts, categorised as either a strong positive correlation or contextual dependence. The results are summarised in Table 3 and illustrates that an overwhelming number of mentors( *n*=19) believed in a strong positive relationship between mentorship and the development of clinical competence. One mentor noted that positive mentorship depended on various factors, indicating that mentorship effectiveness may vary. Therefore, initiatives aimed at enriching mentoring engagement for students in clinical settings supported by dedicated mentors should receive robust support, including appropriate resources and training.

**Table 3.** Perceived Relationship between mentorship and clinical competence.

Correlation	Number of mentors ( *n*=20)
Strong Positive influenced Perceived	19
Dependent on Contextual Dependence	1

The findings demonstrated a strong preference among mentors for daily interactions, reflecting their belief in the significance of frequent and consistent engagement. Regular interactions enable continuous feedback, fostering incremental skill development and sustained confidence growth. ^28^ The preference for frequent interactions supports the literature that advocates regular mentor engagement for optimal skill development and professional socialisation. ^29^ Additionally, the flexibility indicated by mentors highlighted the need to accommodate students’ individual learning styles and clinical needs.

These findings suggest that nursing programmes should structure mentorship schedules to balance regularity with flexibility, allowing personalised interactions while maintaining consistent engagement and feedback opportunities to maximise student learning outcomes.

### 4.4 How can strengths and opportunities be leveraged to enhance the mentoring scheme in this study’s clinical setting?

**Figure 1.**
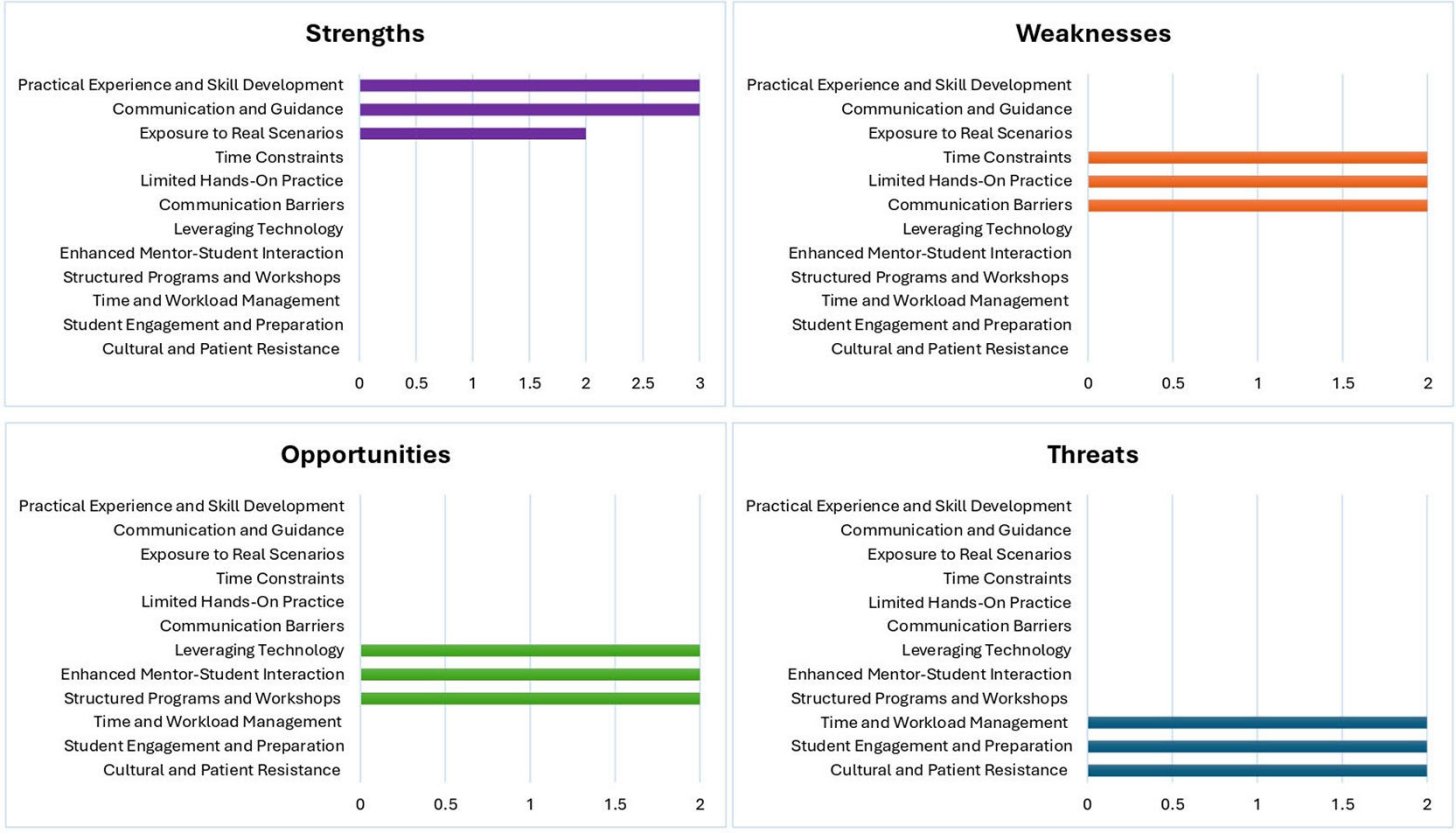
Results of the SWOT analysis. This figure illustrates the strengths, weaknesses, opportunities, and threats associated with the structured clinical mentorship programme. Strengths included practical exposure, communication, and skill development. Weaknesses involved time constraints and communication gaps. Opportunities focused on technological integration, structured mentor engagement, and programme expansion. Threats included workload pressures, variable student engagement, and cultural barriers within diverse clinical environments.

Data related to strength, weaknesses, opportunities and threats were derived from participants responses to specific questions during the semi- structured interviews. These responses were coded inductively and subsequently organized into SWOT categories based on their conceptual alignment. The frequency of responses within each category was calculated to provide a descriptive overview and the values were used to generate Figure 1.

The Figure 1 is intended to complement the qualitative findings rather than represent quantative analysis.

As revealed by the SWOT analysis, the strengths of the current mentoring scheme include the effective integration of practical experience and skill development and linking theoretical learning with real-world applications. Robust communication channels between mentors and mentees ensure timely feedback and support, and exposure to real-world scenarios enhances participants’ adaptability and competence. However, the analysis also pointed out significant weaknesses such as time constraints that could hinder engagement quality, a gap between theoretical instruction and practical application, and communication barriers that may lead to misunderstandings.

Opportunities for enhancing the mentoring process were evident, including leveraging technology to improve accessibility and efficiency, deepening mentor–mentee interactions, and developing structured programmes and workshops to address specific educational needs. However, threats such as inadequate time and workload management, variable student engagement levels, and cultural resistance in diverse settings require strategic management to prevent adverse effects. By addressing these weaknesses and threats while capitalising on opportunities, a mentoring programme can significantly enhance its reach and impact, ultimately improving the overall effectiveness and experience of mentors and mentees.

In addition to the SWOT analysis, we invited the participants to provide additional comments to enhance the mentoring processes within the research context. We conducted a thematic analysis of these comments and identified four main themes that emerged as key areas for further consideration from the mentors’ perspectives.

*4.4.1 Programme duration and structure*

The participants identified the need to revise the duration and structure of the mentoring programme to better align with educational goals and enhance learning outcomes.

*“Extending the duration of the mentorship programme from 20 days to at least 1–2 months to ensure proper exposure and experience”. (M3, M15)*

*“Recommendations for students to work with multiple mentors to gain diverse experiences and perspectives”. (M5)*

The suggested adjustments to the programme duration and structure could provide students with more opportunities to engage with a broader array of mentors, thereby enhancing the overall effectiveness of the mentoring scheme.

*4.4.2 Hands-on clinical learning and engagement*

The participants overwhelmingly emphasised the need for enhanced hands-on clinical learning and engagement.

*“Emphasis on allowing students to perform minor procedures under supervision to gain practical experience”. (M6)*

*“Need for more opportunities for hands-on clinical learning under guidance without other patient responsibilities”. (M12)*

*“Suggestions for dedicated clinical instructors to guide and engage students”. (M14, M16)*

Ensuring robust mentorship and practical experiences is crucial for equipping students with the skills necessary for professional practice.

*4.4.3 Mentor preparation and collaboration*

The participants stressed the importance of a more comprehensive orientation for mentors and effective collaboration with clinical instructors to enhance educational outcomes.

*“Need for proper orientation for mentors regarding their roles and student expectations”. (M2)*

*“Emphasis on improving collaboration between mentors and clinical instructors”. (M8)*

*“Suggestion to ensure mentors are well prepared and committed, enabling growth and serving as role models”. (M20)*

The results indicated that these measures are crucial for ensuring that mentors are well prepared and that there is cohesive support for students’ learning experiences.

*4.4.4 Student accountability and integration*

The participants recognised the importance of student accountability and its integration into decision-making processes.

*“Implement attendance tracking for students, including break times”. (M2)*

*“Ensuring students spend regular time with mentors to decide and meet objectives”. (M7)*

*“Recommending the education of patients about student involvement to increase acceptance”. (M8)*

Drawing on the analysed data, leveraging the identified strengths and opportunities can significantly enhance mentoring schemes in clinical settings. Extending mentorship programmes to one or two months can deepen engagement and improve mentoring relationships. Integrating diverse mentors and practical activities into the curriculum can enrich learning experiences and effectively blend theoretical knowledge with practical skills. Comprehensive mentor orientation and clear student accountability measures are crucial for maintaining mentorship quality.

Students should fully engage in these opportunities for professional growth by focusing on acquiring practical skills and engaging in professional conduct. Clinical mentors and sites must prepare for extended mentoring periods, diversify their experience, and invest in mentor training. Regular monitoring and patient education initiatives can improve mentorship quality and patient outcomes, fostering a dynamic and effective mentoring environment that benefits the entire healthcare community.

The SWOT analysis revealed strengths, including the effective integration of practical experience, robust mentor–mentee communication, and exposure to real-world clinical scenarios. Mentors suggested leveraging opportunities such as incorporating technology, extending programme duration, and enhancing structured mentor–student interactions. However, significant weaknesses such as time constraints, communication barriers, and inconsistent student engagement were identified. This aligns with global findings suggesting that structured mentorship frameworks are the best practices for enhancing clinical education. ^27^ These implications highlight the need for strategic interventions to address the identified weaknesses and leverage strengths and opportunities. Nursing leaders should consider providing structured and protected mentorship time, enhancing mentor orientation, implementing technological solutions for better communication, and increasing hands-on clinical opportunities. This holistic approach is likely to enhance mentorship effectiveness by benefiting both students and mentors.

## 5. Conclusions

The research conducted provides qualitative insight into mentors’ views on mentorship in relation to the culture of the United Arab Emirates. According to the results, mentorship is seen by the mentors as a crucial component that can assist nurses to progress through their learning, gain confidence and go through transformation of their theoretical knowledge into practice.

Specific mentoring skills mentioned by the participants as crucial for creating an atmosphere beneficial to learning include the skills to effectively communicate, be patient, empathic and sensitive to culture. In addition, structured interaction between mentors and student was stated as important for engagement in clinical learning.

It is worthwhile to note that in spite of the significance of this issue, the research does not provide evidence of the impact of mentoring on the learning process based on any measurable parameters, such as competence, confidence and effectiveness of students. The findings provided are, thus, based solely on mentors’ perceptions.

Some recommendations about improvements in mentoring were made by the participants of the research as well, including mentoring preparation, mentoring program design and use of technology in mentoring activities. These recommendations are not validated within the framework of this study.

In summary, the findings contribute to a contextually grounded understanding of mentorship within multicultural health care environments and highlight the importance of culturally responsive and well supported mentorship frameworks in nursing education.

Moreover, addressing the structural barriers and leveraging strategic enhancements are essential for optimising the effectiveness of mentorship programmes. Several strategic recommendations are provided to strengthen mentorship practices and the quality of clinical education.

1. Based on the mentors’ perspectives, participants suggested that health care institutions should consider addressing structural barriers by establishing protected mentorship time and managing workload to facilitate meaningful mento- student interactions. ^1, 26^
2. Participants highlighted the importance of mentor preparation and suggested structured training programs focussed on should be prioritised, focusing on essential skills such as communication, patience, empathy and cultural sensitivity and mentorship skills to enhance the effectiveness of mentorship practices. ^9, 28^
3. Participants suggested that nursing education programmes should integrate innovative digital learning platforms, including virtual and metaverse-based mentoring approaches, to facilitate immersive, flexible, and interactive mentoring experiences. Technological integration will effectively address traditional mentorship limitations, improve engagement, and enhance the overall quality of clinical education quality. ^4, 23^
4. Participants emphasized the value of a closed collaboration between nursing schools and clinical institutions and suggested ongoing support to mentors may contribute to smoother transition to clinical practice and reduce potential errors. ^17^
5. Participants suggested that mentorship duration and structure should be reassessed to better align with extended mentorship periods thereby supporting deeper engagment and skill development. Furthermore, they indicated dedicated meeting times, interactive workshops and more structured programs may enhance mentorship effectiveness and better support student preparedness. ^8, 9, 25^
6. Participants emphasised the relevance of addressing language and cultural factors in mentorship relationships and suggested the inclusion of cultural competency training into mentorship preparation programmes to improve communication effectiveness and foster inclusive learning environments. ^5, 13, 14^

Implementing these recommendations promises substantial improvements in mentorship quality and enhances nursing education and professional practice standards. Ultimately, strategic attention to mentorship structures, mentor preparation, technological innovation, and cultural responsiveness will contribute profoundly to developing competent, confident, and culturally informed nursing professionals in the UAE and beyond.

## 6. Limitations, and implications

### 6.1 Limitations

Several limitations should be considered when interpreting the findings of this study:

• The study was conducted among mentors from selected hospitals in the UAE and may not reflect all clinical settings or specialties.
• Data were self-reported and may be influenced by social desirability or recall bias.
• Only mentors’ perspectives were explored; including students, clinical instructors, and administrators could provide a more holistic understanding.
• The cross-sectional nature of data collection limits the ability to capture changes in mentorship practices over time.

Despite these limitations, the study offers valuable insights into structured mentorship within multicultural healthcare settings.

### 6.2 Implications for nursing practice

Based on the mentors’ perspectives, participants suggested that health care insituitions should consider establishing protected mentorship time to promote consistent and meaningful student mentor interactions.

Structured Participants also emphasized the importance of structured mentorship approaches within clinical practice to support student learning.

Mentors highlighted the potential value of ongoing professional development focussing on communication skills, cultural sensitivity and feedback strategies to enhance mentorship effectiveness.

### 6.3 Implications for nursing education

Participants suggested that nursing education programs may benefit from integrating structured mentorship models within clinical curricula to support continuity between theory and practice.

Strengthening collaboration between academic instituitons and clinical settings was also identified as important for aligning expectations and enhancing student preparedness.

Mentors further indicated that technology enhanced approaches, such as digital learning platforms and simulation based tools, could complement traditional mentorship and lead to further student engagement. ^31^

### 6.4 Implications for Health policy and Leadership

From a policy perspective, participants highlighted the potential importance of mentorship as a strategic component of workforce development

Mentors suggested mentorship frameworks and mentor preparation initiatives supported through instituitional and regulatory mechanisms.

Institutional investment in digital infrastructure was identified as a potential enabler for scalable and flexible mentorship practices.

### 6.5 Implications for future research

Future studies should incorporate perspectives from students, nurse educators, and clinical leaders to capture a more comprehensive view of mentorship.

Longitudinal studies are recommended to explore how mentorship practices influence student development

Further studies are recommended to explore the role of digital and AI-supported mentorship approaches in enhancing clinical education.

In summary, structured mentorship is a critical component of clinical nursing education and has significant implications for nursing practice, education, policy, and research in the UAE. Strengthening mentorship frameworks through cultural responsiveness, organisational support, and technological integration can advance nursing education and contribute to the development of a competent and confident nursing workforce.

## Data Availability

Participant data contains sensitive personal information, and sharing such data publicly could compromise confidentiality and anonymity. The Institutional Review Board (IRB) at the University of Sharjah has mandated that data sharing is permissible only under specific conditions that ensure participant privacy and align with ethical guidelines. Access to the data may be granted to qualified researchers for legitimate academic purposes upon request. Requests for access must be submitted in writing to the principal author, Dr. Jacqueline Maria Dias, jdias@sharjah.ac.ae

## References

[1] Mathisen C Bjørk IT Heyn LG : Practice education facilitators’ perceptions and experiences of their role in the clinical learning environment for nursing students: A qualitative study. *BMC Nurs.* 2023;22(1):165. 10.1186/s12912-023-01328-3 37198631 PMC10189689

[2] Tuomikoski AM Ruotsalainen H Mikkonen K : Nurses’ experiences of their competence at mentoring nursing students during clinical practice: A systematic review of qualitative studies. *Nurse Educ. Today.* 2020;85:104258. 10.1016/j.nedt.2019.104258 31830638

[3] Khowaja AA Dias JM : Students’ perspectives regarding clinical preceptors (CPs) in the baccalaureate undergraduate nursing programme in Karachi, Pakistan. *Scholarship of Teaching and Learning in the South.* 2019;3(1):26–35. 10.36615/sotls.v3i1.68

[4] Kim Y Kim MY : Effects of metaverse-based career mentoring for nursing students: A mixed methods study. *BMC Nurs.* 2023;22(1):160. 10.1186/s12912-023-01323-8 37183255 PMC10183309

[5] Dias JM Aderibigbe S Abraham MS : Undergraduate nursing students’ mentoring experiences in the clinical practicum. *J. Nurs. Manag.* 2022;30:4304–4313. 10.1111/jonm.13833 36193552

[6] Schuler E Mott S Forbes PW : Evaluation of an evidence-based practice mentorship programme in a paediatric quaternary care setting. *J. Res. Nurs.* 2021;26(1–2):149–165. 10.1177/1744987121991417 35251236 PMC8894771

[7] Viera CA : A comparison of mentoring and coaching: What’s the difference?. *Perform. Improv.* 2021;60(7):13–20. 10.1002/pfi.21993

[8] Djiovanis SG : Effectiveness of formal mentoring on novice nurse retention: A comprehensive literature review. *J. Nurses Prof. Dev.* 2023;39(4):E66–E69. 10.1097/NND.0000000000000838 35025831

[9] Lillekroken D Kvalvaag HM Lindeflaten K : Educating the nurses of tomorrow: Exploring first-year nursing students’ reflections on a one-week senior peer-mentor supervised inspiration practice in nursing homes. *BMC Nurs.* 2024;23(1):132. 10.1186/s12912-024-01768-5 38378512 PMC10877788

[10] Cross M Lee S Bridgman H : Benefits, barriers, and enablers of mentoring female health academics: An integrative review. *PLOS One.* 2019;14(4):e0215319. 10.1371/journal.pone.0215319 30998791 PMC6472752

[11] Hussein AHM Taha EE Shalaby SA : Validating mentorship in nursing education: An Egyptian perspective. *Springer eBooks.* 2023; pp.479–486. 10.1007/978-3-031-25204-4_65

[12] Frøiland CT Husebø AML Akerjordet K : Exploring mentorship practices in clinical education in nursing homes: A qualitative mixed-methods study. *J. Clin. Nurs.* 2022;31(7–8):895–908. 10.1111/jocn.15943 34278645

[13] Wang Y Hu S Yao J : Clinical nursing mentors’ motivation, attitude, and practice for mentoring and factors associated with them. *BMC Nurs.* 2024;23(1):76. 10.1186/s12912-024-01757-8 38287369 PMC10826088

[14] Challinor J : Global oncology nursing recruitment and retention: A SWOT analysis. *Semin. Oncol. Nurs.* 2023;39(1):151361. 10.1016/j.soncn.2022.151361 36621414

[15] Mazibu A Downing C Rasesemola R : Expatriate professional nurses’ experiences of preceptorship in a tertiary hospital in Saudi Arabia. *Saudi Journal for Health Sciences.* 2024;13(1):14–20. 10.4103/sjhs.sjhs_150_23

[16] Joseph HB Issac A George AG : Transitional challenges and role of preceptor among new nursing graduates. *J. Caring Sci.* 2022;11(2):56–63. 10.34172/jcs.2022.16 35919276 PMC9339132

[17] Kennedy A : Nurse preceptors and preceptor education: Implications for preceptor programs, retention strategies, and managerial support. *Medsurg Nurs.* 2019;28(2):107–113. Reference Source

[18] Jochim V Rosengren K : Nursing preceptorship, a supportive and reflective approach for promoting a healthy working environment: A multi-methods design. *Nordic Journal of Nursing Research.* 2021;42(3):147–157. 10.1177/20571585211025207

[19] Loughran MC Koharchik L : Ensuring a successful preceptorship. *Am. J. Nurs.* 2019;119(5):61–65. 10.1097/01.NAJ.0000557917.73516.00 31033558

[20] Cao X Li J Gong S : The relationships of both transition shock, empathy, resilience, and coping strategies with professional quality of life in newly graduated nurses. *BMC Nurs.* 2021;20(1). 10.1186/s12912-021-00589-0 PMC8062214 33888101

[21] Mikkonen K Tomietto M Tuomikoski A : Mentors’ competence in mentoring nursing students in clinical practice: Detecting profiles to enhance mentoring practices. *Nurs. Open.* 2021;9(1):593–603. 10.1002/nop2.1103 34726336 PMC8685782

[22] Gularte-Rinaldo J Baumgardner R Tilton T : Mentorship respect study: A nurse mentorship program’s impact on transition to practice and decision to remain in nursing for newly graduated nurses. *Nurse Lead.* 2023;21(2):262–267. 10.1016/j.mnl.2022.07.003 35990373 PMC9375843

[23] Frøiland CT Husebø AML Aase I : A digital educational resource to support and enhance effective mentorship practices of nursing students in nursing homes: A qualitative study. *BMC Nurs.* 2023;22:423. 10.1186/s12912-023-01570-9 37953235 PMC10641992

[24] Erdal NU : The effect of mentoring on the performance of nurses in developing career and psychosocial functions. *Asian Journal of Advances in Medical Science.* 2022;4(1).

[25] Elo S Kyngäs H : The qualitative content analysis process. *J. Adv. Nurs.* 2008;62(1):107–115. 10.1111/j.1365-2648.2007.04569.x 18352969

[26] Braun V Clark V : Using thematic analysis in psychology. *Qualitative Research in psychology.* 2006;3(2):77–101. 10.1191/1478088706qp063oa

[27] Blake-Beard S Shapiro M Ingols C : A model for strengthening mentors: Frames and practices. *Int. J. Environ. Res. Public Health.* 2021;18(12):6465. 10.3390/ijerph18126465 34203753 PMC8296284

[28] Weinberg FJ : How and when is role modeling effective? The influence of mentee professional identity on mentoring dynamics and personal learning outcomes. *Group Org. Manag.* 2019;44(2):425–477. 10.1177/1059601119838689

[29] Dias JM Al Kaabi FSBS Al Hooti ST : Exploring peer tutoring experiences in improving nursing students’ academic success and performance: A qualitative study among Emirati undergraduate nursing students. *F1000Research.* 2025;14:1416. 10.12688/f1000research.172023.1 41509105 PMC12775669

[30] Mottershead R Alonaizi N : Empowering social prescribing and peer support: A proposed therapeutic alliance against addiction and substance misuse within the Middle East. *J. Drug. Alcohol. Res.* 2022;11:97203.

[31] Dias JM Abraham MS Sub MA : Exploring simulation as a teaching pedagogy for male undergraduate nursing students: A qualitative study in the United Arab Emirates. *F1000Research.* 2025;14:920. 41195157 10.12688/f1000research.167350.2 PMC12583910

