# Supplementary material for: Mentor’s Perspective on Structured Clinical Mentoring in the Arab Context
Source: F1000Res. 2026 Apr 30;15:432. Originally published 2026 Mar 25. [Version 2] doi: 10.12688/f1000research.177515.2 (PMC13146465; doi:10.12688/f1000research.177515.2)
Supplement: Peer review history

# Reviewer report: Reviewer response for version 2

**DOI:** 10.5256/f1000research.198750.r480580

**Published:** 2026-05-05

**Authors:** Syed Fahad Javaid

**Affiliations:** 1 United Arab Emirates University, Al Ain, United Arab Emirates

Thank you very much for considering my comments and amending the article accordingly. The changes have improved the academic value of this paper, and I wish the authors all the best.

Is the work clearly and accurately presented and does it cite the current literature?

Partly

If applicable, is the statistical analysis and its interpretation appropriate?

Partly

Are all the source data underlying the results available to ensure full reproducibility?

No

Is the study design appropriate and is the work technically sound?

Partly

Are the conclusions drawn adequately supported by the results?

Partly

Are sufficient details of methods and analysis provided to allow replication by others?

No

Reviewer Expertise:

Medical education, student perceptions, support systems.

I confirm that I have read this submission and believe that I have an appropriate level of expertise to confirm that it is of an acceptable scientific standard.

# Reviewer report: Reviewer response for version 1

**DOI:** 10.5256/f1000research.195749.r470796

**Published:** 2026-04-11

**Authors:** Syed Fahad Javaid

**Affiliations:** 1 United Arab Emirates University, Al Ain, United Arab Emirates

Thank you for the opportunity to read and review this study that explores mentor perspectives from 20 participants involved in final clinical placements. This manuscript addresses an underexplored topic that is mentors’ perspectives on structured clinical mentoring in nursing education within the regional context. The topic is relevant, the regional focus is potentially valuable. The use of verbatim quotations is helpful, and the SWOT framework is good. The overall descriptive contribution is acceptable. The problem is that the manuscript repeatedly moves beyond what the data can actually support. The evidence presented is limited to self-reported mentor perceptions, yet the paper frequently frames its conclusions as if it had demonstrated actual effects on student competence, confidence, and readiness for practice. My recommendation is Major Revision. My major concerns are as below:

 1. There exists a basic scientific reporting issue. The abstract refers to “structured and semi-structured in-depth interviews” and an “inductive thematic approach,” while the methods section refers to “structured interviews” and “qualitative content analysis.” The analysis section then cites Graneheim and Lundman content analysis. These are not interchangeable descriptions. At present, it is unclear whether this was a structured interview study, a semi-structured interview study, a thematic analysis, or a content analysis study.

 2. In its current form, there are insufficient details to reproduce the study. The interview guide is only summarised in broad topic areas. The exact interview questions are not provided. There is also no adequate participant description beyond the fact that there were 20 mentors. The paper should report, at minimum, participant characteristics such as years of experience, clinical area/speciality, mentor training, role type, and relevant demographic/contextual information. The setting is also vague: the manuscript refers both to a “semi-public university” and to hospitals across the UAE but does not clearly define the recruitment frame or institutional context.

 3. The paper states that interviews continued until data saturation was reached, but it does not explain how saturation was defined, who judged it, how it was documented, or at what point no new codes/themes emerged. This is especially important in a qualitative study with a modest sample size. A statement of saturation without procedural detail is not enough.

 4. The manuscript says two researchers independently coded the data, but it does not explain how codes were generated, whether a codebook was developed, how disagreements were resolved, whether coding was iterative, or how final themes were derived. The same problem applies to the SWOT analysis and Figure 1…. the paper does not explain fully how comments were assigned to SWOT categories or how the bar values were produced.

 5. This is presented as a qualitative study, yet the paper introduces tables with percentages such as 60%, 35%, and 95%. With a sample of 20, those figures are potentially problematic. More importantly, Table 3 is labelled as showing a ***“correlation between mentorship and clinical competence,” *** but no correlation analysis was performed. The table only reports how many mentors believed there was a strong positive relationship. That is not a correlation. This terminology is incorrect and should be removed. If the authors want to retain numerical summaries, they should use raw counts and explicitly frame them as descriptive tallies within a qualitative study, not statistical evidence.

 6. The conclusions overstate what the data show. The study does not directly measure student competence, confidence, readiness for practice, or clinical outcomes. It only reports mentors’ perceptions of those issues. Therefore, statements such as mentorship “significantly influenced” students’ clinical skills and that mentoring structure/frequency “directly impact” learning are stronger than the evidence warrants. There are no outcome measurements mentioned.

 These should be rewritten as perceived influences reported by mentors. The same caution applies to the recommendations about technology, programme duration, and broader policy implications. These may be reasonable suggestions, but they are not directly established by the presented data.

 7. There is duplication and redundancy in text…for instance the paper contains both a “5. Conclusions” section and then a second “6. Conclusion, limitations, and implications/6.1 Conclusion,” which is redundant and poorly structured.

Is the work clearly and accurately presented and does it cite the current literature?

Partly

If applicable, is the statistical analysis and its interpretation appropriate?

Partly

Are all the source data underlying the results available to ensure full reproducibility?

No

Is the study design appropriate and is the work technically sound?

Partly

Are the conclusions drawn adequately supported by the results?

Partly

Are sufficient details of methods and analysis provided to allow replication by others?

No

Reviewer Expertise:

Medical education, student perceptions, support systems.

I confirm that I have read this submission and believe that I have an appropriate level of expertise to state that I do not consider it to be of an acceptable scientific standard, for reasons outlined above.

# Reviewer report: Reviewer response for version 1

**DOI:** 10.5256/f1000research.195749.r470790

**Published:** 2026-04-01

**Authors:** Haitham Khatatbeh

**Affiliations:** 1 Yarmouk University, Irbid, Irbid Governorate, Jordan

Dear Authors,

 The study is academically meritorious and provides valuable regional insights. However, I have some comments:

 * While a sample size of 20 is standard in qualitative study, the "Results" section should briefly mention at which interview thematic saturation was reached (e.g., "No new themes emerged after the 15th interview"). This proves the sample size was sufficient and not arbitrary.

 * The "Research Instrument" section is detailed, listing the three sections of the interview guide. However, to allow for full replication, the exact interview questions should be included as an appendix.

 *While the themes are supported by vignettes (e.g., M1, M4), the full "source data" (anonymized transcripts) is not explicitly linked or made available via a repository, which is increasingly a requirement for full reproducibility.

 * The integration of a SWOT framework (Figure 1) is a strong analytical choice for "Research Question 4," as it translates qualitative feedback into actionable strategic categories.

 *The conclusion regarding "culturally informed mentorship" is supported by the literature review (Section 2.1) and the "Patience and Understanding" theme (Section 4.2.2). However, more specific examples of how UAE culture influenced specific clinical decisions would have strengthened the link further.

 * The authors use percentages (e.g., "95% believed...") for a sample of only 20 people. In qualitative research, these figures can be misleading as they imply statistical significance that doesn't exist. Rephrase these as "19 out of 20 participants" rather than "95%" to maintain the qualitative integrity of the study.

 In conclusion, the work is of high quality and suitable for indexing, provided the authors perform a final proofread for minor grammatical consistency and consider attaching the full interview guide.

Is the work clearly and accurately presented and does it cite the current literature?

Yes

If applicable, is the statistical analysis and its interpretation appropriate?

I cannot comment. A qualified statistician is required.

Are all the source data underlying the results available to ensure full reproducibility?

No

Is the study design appropriate and is the work technically sound?

Yes

Are the conclusions drawn adequately supported by the results?

Partly

Are sufficient details of methods and analysis provided to allow replication by others?

Yes

Reviewer Expertise:

Nursing Education, pediatric nursing, public health

I confirm that I have read this submission and believe that I have an appropriate level of expertise to confirm that it is of an acceptable scientific standard.
